# Comparison of DBFS with MoCA and MMSE tools for MCI screening

**DOI:** 10.6026/97320630019522

**Published:** 2023-05-31

**Authors:** Eun Yip Chad Chew, Pillay Prem, Kuah Jasmine, Vij Nav, Balasundaram Arthi

**Affiliations:** 1 Neurowyzr Pte. Ltd., 6 Raffles Quay, 11-07, Singapore-048580

**Keywords:** Mild cognitive impairments, MoCA, MMSE, DBFS tool, comparison

## Abstract

Mild cognitive impairment (MCI) has been associated with many diseases. The MCI could be a marker for the early diagnosis of certain diseases. Early detection of MCI could be beneficial for restoration of cognitive reserves. One hundred and five subjects were included in the study, underwent the Digital Brain Function Screen (DBFS) test as well as the Montreal Cognitive Assessment (MoCA) test and 73 subjects took the Mini-Mental State Examination (MMSE) test. DBFS test and retest was taken by 16 subjects. The test scores of DBFS tool showed significant positive correlation with MoCA and MMSE test scores. In conclusion, the DBFS tool could be an effective digital tool which can overcome the disadvantages of traditional tools of screening MCI like MoCA and MMSE.

## Background:

Mild cognitive impairment (MCI) is regarded as the transitional period between the normal cognitive decline of healthy ageing and dementia. Some of the risk factors for developing MCI include diabetes, depression, and stroke [1, 2,3,4]. The MCI prevalence ranged from 7% to 25% among the older population of age ranging from 60-85 years [5]. Approximately more than 40% older adults with MCI had underlying AD pathology [6]. Furthermore, studies have stated that an estimated 10 to 20% of people aged 65 or older with MCI develop dementia over a one-year period [7]. However, not everyone who has MCI develops dementia. In many cases, the symptoms of MCI may stay the same or even improve. Hence, early screening for MCI is crucial for recovering the cognitive reserves in individuals both healthy and with other underlying reasons [8]. To assess cognitive functions, there have been many traditional tests which are pen and paper tests. These may include Mini-Mental State Examination (MMSE) and the Montreal Cognitive Assessment (MoCA) tests [9,10]. These both pen and paper tests are time consuming, require healthcare training and expertise, and costly which may contribute to their limitation of these tests [11]. The present study introduces a user-friendly digital cognitive screening test called the "DBFS" developed for the detection and monitoring of cognitive decline and impairment in adults. In this clinical study, we compared the accuracy of our newly developed digital cognitive screening test, the "DBFS", against the MoCA and MMSE. We also analysed how the "DBFS" correlates to traditional cognitive screening tools, MoCA and MMSE.

## Subjects and Methods:

This prospective comparison trial was conducted at Singapore Brain Spine Nerves Centre and initiated after obtaining the ethical approval (No: CNDBFSCV0012021). The DBFS test for screening MCI was employed in the study along with other tools like MoCA and MMSE. A total of 105 participants completed the "DBFS" and MoCA, while a 73 of total participants completed the MMSE. All the participants have provided informed consent before the initiation of the study. All neurologically healthy participants aged between 12 to 85 years were included in the study. Those subjects with age less than 12 years or with central neurological deficits were excluded from the study. The study participants were selected based on consecutive sampling. The study test DBFS was compared to reference tests like MoCA and MMSE for assessing reliability and replication. Sixteen of the participants who were recruited from the brain and spine clinic repeated the "DBFS" as part of their brain health monitoring process. The "DBFS" test also provides domain scores in four cognitive domains - immediate memory, working memory, attention, and executive function. These domains will be flagged out if the domain scores fall below one standard deviation (scores of 84 and below). Participants who had a normal overall "DBFS" score but had one or more of their cognitive domains flagged out and scored below 26 for MoCA were categorised as MCI for their "DBFS" score [10].

## Statistical analysis:

An average of the "DBFS" test scores were used for this research's analysis. Scores from their first "DBFS" test ("DBFS" t1) and second "DBFS" test ("DBFS" t2) were used to calculate the test-retest reliability of "DBFS" test. Analysis was conducted with Statistical Package for the Social Sciences 27.0.0 package (SPSS, 2020). Pearson correlation test was done to obtain the correlation and significance of both the tests. The Bland-Altman test was performed between the MoCA and DBFS test.

## Results:

The study participants included 56 male and 49 female. The age groups of the participants are listed in Table 1. There was a significant and positive correlation between the "DBFS" overall scores and MoCA scores (r = 0.62, p<.01), as shown below in Table 2.

## Correlation between the study tests:

The test-retest-reliability coefficient was found to be high (r = 0.74, p<.01), as shown below in Table 3, suggesting good reliability of the DBFS test scores. The MoCA and MMSE have been found to have a significant moderate correlation (r = 0.51, p<.01), as shown in Table 4.

## Agreement between the two tests:

**Figure 1:**
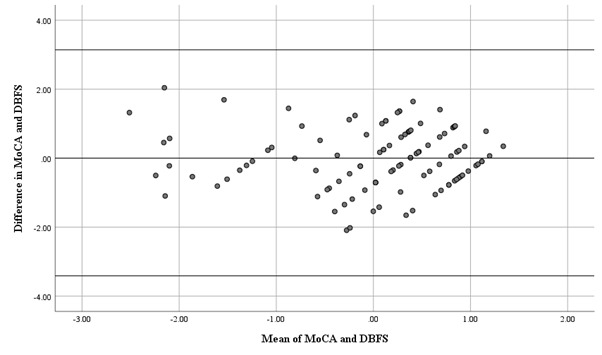
Bland-Altman plot of standardized test scores

Bland-Altman plot with standardised values showing that 100 % of data points lie within ±2SD of the mean difference, as shown in Figure 1

## Discussion:

This prospective study determines the non-inferiority of the DBFS test in comparison to both MoCA and MMSE. Between the MoCA and the MMSE, the correlation is similarly moderate indicating that both tests are suitable for use in the detection of cognitive decline [12]. However, even though the MMSE is a suitable widely used cognitive screening tool, it has been shown that the MoCA is far more superior to MMSE in multiple study settings, as the MMSE had a lower sensitivity in the detection of MCI [12,13,14, 15]. These previous study inferences are in line with outcomes of our study product. The bias of the study product scores was minimal and in agreement as revealed by the Bland-Altman plot test using the mean differences between the DBFS test scores and MoCA test scores [16]. In summary, there was a statistically significant correlation found between "DBFS" and MoCA scores and a significant 97.1% match of clinical outcomes between the "DBFS" and MoCA. The "DBFS" was able to achieve a sensitivity of 86.4% and specificity of 100% in detecting MCI. Adding on, "DBFS" was able to distinguish between individuals with MCI from healthy ones with adequate accuracy.

## Conclusion:

The "DBFS" has been found to be a good digital substitute of the gold standard MoCA, having had a statistically high match in terms of clinical outcomes. Moreover, the "DBFS" has certain advantages over the traditional pen-and- paper MOCA. For instance, this test can be self-administered or assisted by a nonprofessional staff or family member, which makes the "DBFS" a useful tool for case-finding in primary healthcare and community settings. More importantly, the "DBFS" serves as an important tool in spearheading early efforts for detecting MCI in the general healthy population, as part of a preventative approach towards cognitive decline. It is an excellent tool for the primary health physician carrying out health screening including executive health screening as well as for the busy specialist. This low-cost, clinically validated digital test can be used for both in-person medical visits as well as for telemedicine consults.

## Funding sources:

This research received no specific grant from any funding agency in the public, commercial or not-for-profit sectors.

## Figures and Tables

**Table 1:** Study participants age distribution

**Age group (years)**	**Frequency (n)**
13-20	6
21-30	13
31-40	31
41-50	27
51-60	11
61-70	14
71 and above	3

**Table 2:** Pearson Correlation between DBFS test scores and MoCA scores

**Variable**		**n**	**M**	**SD**	**1**	**2**
1	DBFS test	105	118.28	24.99		0.62**
2	MoCA	105	27.05	2.54	0.62**	
**Correlation is statistically significant at p<0.01

**Table 3:** Test-retest reliability of DBFS test

**Variable**		**n**	**M**	**SD**	**1**	**2**
1	DBFS test t1	16	116.08	35.61		0.74**
2	DBFS test t2	16	117.63	26.66	0.74**	
**Correlation is statistically significant at p<0.01

**Table 4:** Pearson Correlation between MoCA and MMSE scores

**Variable**		**n**	**M**	**SD**	**1**	**2**
1	MoCA	73	26.92	2.71		0.51**
2	MMSE	73	29.12	1.6	0.51**	
**Correlation is statistically significant at p<0.01
